# Pretreatment CRP–Albumin–Lymphocyte (CALLY) Index as a Prognostic Biomarker of Survival and Recurrence‐Free Survival in Patients With Early‐Stage Cervical Cancer After Radical Hysterectomy: A Multicenter Retrospective Cohort Study

**DOI:** 10.1155/ogi/6137796

**Published:** 2026-02-02

**Authors:** Mansour Bahardoust, Nasim Ghalavand, Mohammadsadra Shamohammadi, Mina Salehi Asl, Yasaman Tavakoli, Aref Alinejad, Armaghan Abbasi Garavand, Meisam Haghmoradi, Sara Ghorbanzadeh, Adnan Tizmaghz

**Affiliations:** ^1^ Department of Epidemiology, School of Public Health, Shahid Beheshti University of Medical Sciences, Tehran, Iran, sbmu.ac.ir; ^2^ School of Nursing and Midwifery, Ahvaz Jundishapour University of Medical Sciences, Ahvaz, Iran, ajums.ac.ir; ^3^ Gastrointestinal and Liver Diseases Research Center, Iran University of Medical Sciences, Tehran, Iran, iums.ac.ir; ^4^ Department of Cell Biology and Anatomical Sciences, School of Medicine, Shahid Beheshti University of Medical Sciences, Tehran, Iran, sbmu.ac.ir; ^5^ Student Research Committee, Department of Medicine, Mazandaran University of Medical Science, Mazandaran, Sari, Iran, mazums.ac.ir; ^6^ School of Medicine, Iran University of Medical Science, Tehran, Iran, iums.ac.ir; ^7^ Department of Orthopedic Surgery, Urmia University of Medical Sciences, Urmia, Iran, umsu.ac.ir; ^8^ Department of Obstetrics and Gynecology, Iran University of Medical Sciences, Tehran, Iran, iums.ac.ir; ^9^ Department of General Surgery, School of Medicine, Firoozabadi Hospital, Iran University of Medical Sciences, Tehran, Iran, iums.ac.ir

**Keywords:** CALLY, cervical cancer, CRP–albumin–lymphocyte index, recurrence, survival

## Abstract

**Introduction/Background:**

The C‐reactive protein (CRP)–albumin–lymphocyte (CALLY) index is a new prognostic biomarker combining CRP, serum albumin, and lymphocyte count that can be associated with the survival of cancer patients by assessing immune, nutritional, and inflammatory status as an important immune indicator. The association of CALLY index as a marker predicting survival of cancer patients with cervical cancer (CC) remains unclear. This study aimed to evaluate the prognostic value of the CALLY index with overall survival (OS) and recurrence‐free survival (RFS) in patients with early‐stage CC after radical hysterectomy.

**Methodology:**

In this multicenter retrospective cohort study, we examined the medical profile of 806 women with early‐stage CC who underwent Type II/III radical hysterectomy and bilateral pelvic lymphadenectomy at three centers affiliated to our center between 2012 and 2022. The CALLY index was calculated before treatment. OS and RFS were the primary endpoints. Kaplan–Meier and Cox models assessed the association between CALLY index and outcomes, adjusting for age, histology, tumor size, FIGO stage, grade, extent of lymphadenectomy, and adjuvant therapy. A CALLY index cutoff of 3 maximized discrimination (AUC 0.822; 95% CI, 0.75–0.90).

**Results:**

Five‐year OS was higher with CALLY index ≥ 3 vs. < 3 (82.1% vs. 71.2%; log rank *p* = 0.009), as was 5‐year RFS (76.4% vs. 64.2%; *p* = 0.001). Multivariate analysis showed that CALLY index ≥ 3 was independently associated with improved OS (HR 0.87; 95% CI, 0.78–0.96; *p* = 0.001) and RFS (HR 0.86; 95% CI, 0.78–0.95; *p* = 0.001). In addition, age ≥ 45 years, nonsquamous histology, tumor size ≥ 3 cm, FIGO stage > IB, grade > G2, and LNR > 40% were significantly associated with poorer OS and RFS, whereas receiving adjuvant therapy was associated with a better prognosis.

**Conclusions:**

Pretreatment CALLY index is an independent, readily obtainable prognostic biomarker for OS and RFS after radical hysterectomy in early‐stage CC. This index can be useful as a predictor of the prognosis for patients with CC.

## 1. Introduction

Cervical cancer (CC) remains a significant global health burden, accounting for the fourth most prevalent cause of cancer incidence and mortality in women globally [1]. Human papillomavirus infection, particularly high‐risk types 16 and 18, is the primary etiologic factor for CC [1–3]. Additional risk factors include early age at first intercourse, multiple sexual partners, high parity, long‐term oral contraceptive use, and smoking [2, 3]. Although the 2018 FIGO (International Federation of Gynecology and Obstetrics) staging system incorporates lymph‐node status and the tumor–node–metastasis (TNM) classification provides detailed anatomic descriptors, both systems remain primarily anatomic and incompletely reflect tumor biology, leading to heterogeneity in outcomes within the same stage [4–6].

To address these limitations, recent studies have explored prognostic indices to evaluate OS and RFS in CC patients. To evaluate survival and recurrence in CC, several prognostic indices have been proposed, including the platelet‐to‐lymphocyte ratio (PLR), the neutrophil‐to‐lymphocyte ratio (NLR), and the CRP‐to‐albumin ratio [7, 8]. Although these indices provide valuable prognostic information, they also have important limitations. Systemic inflammatory markers such as NLR and PLR are prone to external influences, as they primarily reflect systemic inflammation or infection rather than tumor‐specific processes [9].

The CALLY index is a novel prognostic biomarker that combines CRP, serum albumin, and lymphocyte count to assess immune, nutritional, and inflammatory status in cancer patients [10]. The CALLY index, integrating inflammation, nutrition, and immunity, has been consistently validated in studies as a reliable prognostic biomarker in cancers, including gastric [11], colorectal [12], hepatocellular [13], esophageal [14], ovarian, and breast [15], where higher CALLY index is associated with improved OS.

Although the prognostic value of the CALLY index has been evaluated across several malignancies, to our knowledge, its relevance in early‐stage CC has not been established. The purpose of this multicenter retrospective cohort study is to investigate the predictive influence of the pretreatment CALLY index on OS and RFS in patients with early‐stage CC following radical hysterectomy, providing a unique and useful tool for customized risk stratification and therapeutic decision‐making.

## 2. Methodology

### 2.1. Study Design and Population

We conducted a multicenter retrospective cohort study of women with early‐stage epithelial CC who underwent radical hysterectomy with lymphadenectomy at three tertiary care centers affiliated with Iran University of Medical Sciences and Shahid Beheshti University of Medical Sciences. Medical records and pathology databases of 1211 CC patients treated between 2012 and 2022 were reviewed. Finally, 806 patients met the eligibility criteria and were included in the final analysis.

### 2.2. Ethical Considerations

The Ethics Committee of Iran University of Medical Sciences approved the study protocol, and the study was conducted and reported in accordance with the STROBE guidelines for observational studies.

### 2.3. Patient Selection

Eligible patients were women aged ≥ 18 years with histologically confirmed early‐stage CC (FIGO stages IA–IIA) who underwent primary Type II or III radical hysterectomy with bilateral pelvic lymphadenectomy. Inclusion required the availability of complete follow‐up data and pretreatment laboratory measurements necessary to calculate the CALLY index, including CRP, serum albumin, and lymphocyte count, obtained before the initiation of any oncologic therapy. Patients were excluded if they had received neoadjuvant chemotherapy or radiotherapy before surgery; had incomplete clinicopathological data or were lost to follow‐up; presented with distant metastasis at diagnosis; or had nonepithelial cervical malignancies. Additional exclusions included conditions that could confound systemic inflammatory or immune status, such as concurrent malignancy, immunosuppressive disorders or therapies, active infections, or chronic inflammatory diseases. Additionally, patients with more than 10% missing values in any of the three CALLY index components were excluded from the study.

### 2.4. Data Collection and Variables

Demographic and clinical variables included age at diagnosis, body mass index (BMI), smoking status, family history of cancer, and the presence of comorbid conditions such as diabetes and hypertension. The extent of lymphadenectomy was categorized as pelvic alone or pelvic with para‐aortic. Pathologic assessment was conducted locally in accordance with institutional protocols. Abstracted histopathologic variables included histologic subtype (squamous cell carcinoma, adenocarcinoma, and others), maximal tumor diameter, histologic grade, depth of stromal invasion (≤one‐half vs. > one‐half of cervical stromal thickness), lymphovascular space invasion (when reported), total number of lymph nodes retrieved, and number of positive nodes. The lymph node ratio (LNR) was prespecified as the number of positive nodes divided by the total number of nodes removed and was analyzed dichotomously using a threshold of > 40% versus ≤ 40%.

Pretreatment laboratory values were obtained within 1–2 weeks before surgery, which included the CRP level (mg/L), serum albumin concentration (g/L), and lymphocyte count (per μL) from the complete blood count. All assays were performed in certified hospital laboratories using identical reagent kits and standardized procedures. The CALLY index score was calculated using the following formula:

(1) CALLY index=albumin g/dL×lymphocyte count /μLCRP mg/dL×104.

**Figure 1:**
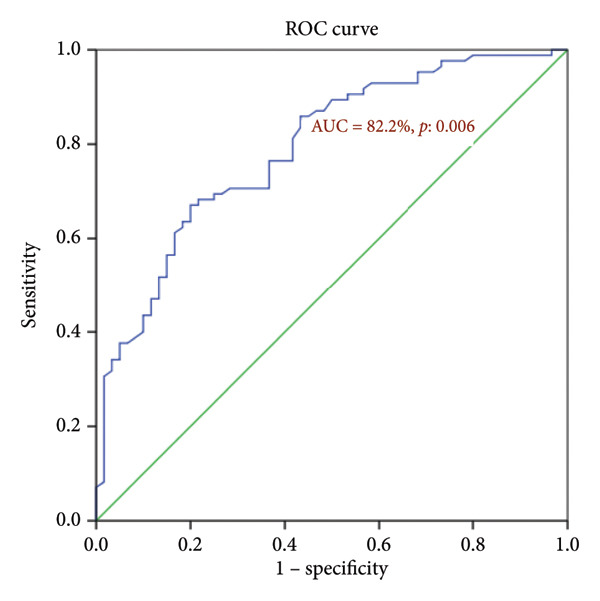
ROC analysis results for the diagnostic accuracy of the CALLY index for survival of patients with CC.

To establish the optimal cutoff value of the CALLY index for prognostic stratification, receiver operating characteristic (ROC) curve analysis was performed. A threshold of 3 was identified, yielding the highest predictive accuracy (area under the curve (AUC) = 0.822, 95% confidence interval (CI) 0.75–0.90) (Figure 1). Based on this cutoff, patients were categorized into two groups for comparative analyses: high CALLY index (≥ 3) and low CALLY index (< 3).

### 2.5. Outcome Measures

The primary endpoints of the study were OS and RFS. OS was defined as the time from the date of radical hysterectomy to the date of death from any cause or last known follow‐up. RFS was defined as the time from surgery to the first documented recurrence of CC. Recurrences were identified through clinical examinations, imaging studies, and/or biopsy confirmation and included any locoregional tumor relapse or distant metastasis.

### 2.6. Statistical Analysis

We used Stata software Version 17 for data analysis. Frequency and % were used to report qualitative variables. Quantitative variables were reported with means and standard deviations. The Kolmogorov–Smirnov test was used to assess the normality of the distribution of quantitative variables in comparison groups. Assuming normality, independent *t*‐tests were used to compare quantitative variables between the two groups; if not, the Mann–Whitney test was used instead. Chi‐squared test was used to compare qualitative variables. Kaplan–Meier analysis was used to estimate OS and RFS, and the difference between the outcome in the two groups (CALLY index < 3 vs. ≥ 3) was compared using the log‐rank test. A univariate Cox proportional‐hazards model was used to assess the association of variables with OS and RFS, and variables that had a *p*‐value less than 0.2 in the univariate analysis test were entered into the multivariate analysis. Cox multivariate analysis was used to assess the association of variables with outcomes (OS and RFS rates) using the backward method. Results were reported as hazard ratios (HRs) and 95% CIs (95% CIs). Variance inflation factors (VIFs) were used to detect the presence of multiple collinearity between variables in the regression model, and a VIFs ≥ 5 was interpreted as collinearity.

A *p* value of less than 0.05 was considered statistically significant.

## 3. Results

Finally, the medical profiles and pathological findings of 806 CC women were reviewed. The mean age of the patients in the all cohort was 46.5 ± 9.8 years. The mean BMI of the patients was 24.9 ± 3.0 kg/m^2^. Of the 806 patients, 73 (9.1%) had a history of smoking. One hundred and thirty‐two (16.4%) had at least one comorbidity. No significant differences were observed for the baseline characteristics of the patients in the two groups (CALLY index ≥ 3 and < 3) (*p* > 0.05). The mean follow‐up period since diagnosis for the patients was 80.1 ± 16.8 months.

The squamous pathological type included more than 80% (660(81.9%)) of the patients. The majority of CC patients were diagnosed at Stages 1B (1 or 2) (60.8%) and IIA (1 or 2) (27.9%). The frequency of patients with higher tumor stages, the depth of primary tumor invasion was more than 1.2, and a higher pathological grade was observed in patients with a CALLY index < 3. No significant differences were observed for other variables between CC patients with a CALLY index score of ≥ 3 and those with a score of < 3. Demographic and tumor characteristics are presented separately by the study group and in general in Table 1.

**Table 1:** Demographic and clinical characteristics of patients at baseline.

Variable	All cohort (N: 806)	CALLY index		*p* value
		< 3 (N: 386)	≥ 3 (N: 420)	
Age median (IQR)	47 (38.56)	47 (37.57)	47 (38.56)	0.58
BMI (kg/m^2^)	24 (22.26)	25 (23.27)	24 (22.26)	0.36
Smoking	73 (9.1%)	35 (9.1%)	38 (9%)	0.88
Family history (positive)	135 (16.8%)	69 (17.9%)	66 (15.7%)	0.52
Comorbidity (positive)	132 (16.4%)	73 (18.9%)	59 (15.3%)	0.082

*Pathologic type*				
Squamous	660 (81.9%)	319 (82.6%)	341 (81.2%)	0.091
Adenocarcinoma	84 (14.1%)	19 (12.8%)	65 (15.5%)	
Others	32 (4%)	18 (4.6%)	14 (3.3%)	

*FIGO stage*				
1A2	91 (11.3%)	34 (8.8%)	57 (13.6%)	0.036
1B (1 or 2)	490 (60.8%)	231 (59.8%)	259 (61.7%)	
IIA (1 or 2)	225 (27.9%)	121 (31.4%)	104 (24.7%)	

*Tumor size (cm)*				
≤ 3	539 (66.9%)	243 (63%)	296 (70.5%)	0.056
> 3	267 (33.1%)	143 (37%)	124 (29.5%)	

*Depth of primary tumor invasion*				
≤ 1/2	499 (61.9%)	247 (64%)	252 (60%)	0.049
> 1/2	307 (38.1%)	139 (36%)	168 (40%)	

*Histological grade*				
G1	85 (10.5%)	40 (10.4%)	45 (10.7%)	0.041
G2	341 (42.3%)	135 (35%)	206 (49.1%)	
G3	380 (47.2%)	211 (54.6%)	169 (40.2%)	

*Lymphadenectomy*				
Pelvic	733 (90.9%)	349 (90%)	384 (91.3%)	0.25
Pelvic + periaortic	73 (9.1%)	37 (10%)	36 (8.7%)	

Adjutant therapy (positive)	331 (41.1%)	154 (39.9%)	177 (42.1%)	0.091
Median follow‐up (month)	81 (67.95)	79 (65.93)	82 (68.96)	0.23

### 3.1. Univariate Analysis

The univariate analysis showed that factors such as age, comorbidities, pathologic type, tumor size, FIGO stage, histological grade, depth of primary tumor invasion, adjuvant therapy, and LNR were significantly associated with both OS and RFS rates in patients with CC (Table 2).

**Table 2:** Predictors of OS and RFS in patients with CC based on the results of univariate analysis.

Variable	OS			RSF		
	5‐year OS rate	HR (95% CI)	*p* value	5‐year RFS rate	95% CI	*p* value
*Age (year)*						
< 45	81.1%	Ref	0.035	75.2%	Ref	0.037
≥ 45	69.7%	1.17 (1.01.1.34)		65.1%	1.16 (1.00.1.34)	

*Smoking*						
No	79.5%	Ref	0.59	74.2%	Ref	0.76
Yes	75.9%	1.05 (0.75.1.31)		69.8%	1.07 (0.65.1.44)	

*Family history*						
No	77.1%	Ref	0.71	72.2%	Ref	0.68
Yes	74.9%	1.03 (0.73.1.31)		68.5%	1.06 (0.70.1.37)	

*BMI (kg/* *m* ^2^ *)*						
< 25	79.4%	Ref	0.15	73.6%	Ref	0.25
≥ 25	74.6%	1.07 (0.9.1.18)		69.2%	1.06 (0.91.1.16)	

*Comorbidity*						
No	79.6%	Ref	0.026	75.2%	Ref	0.022
Yes	69.2%	1.15 (1.02.1.28)		64.3%	1.17 (1.02.1.33)	

*Pathologic type*						
Squamous	81.2%	Ref		76.9%	Ref	
Adenocarcinoma	60.5%	1.35 (1.15.1.55)	0.007	53.2%	1.45 (1.05.1.86)	0.001
Adenosquamous	50.2%	1.62 (1.12.2.13)	0.001	46.5%	1.66 (1.1.2.23)	0.001

*Stage (FIGO)*						
1A2	85.1%	Ref		79.9%	Ref	
1B (1 or 2)	76.5%	1.12 (1.02.1.23)	0.005	70.1%	1.14 (1.03.1.25)	0.018
IIA (1 or 2)	54.2%	1.58 (1.18.2.01)	0.001	48.2%	1.66 (1.16.2.17)	0.004

*Tumor size (cm)*						
≤ 3	86.2%	Ref	0.001	78.9%	Ref	0.012
> 3	62.8%	1.4 (1.11.1.72)		58.3%	1.35 (1.1.1.61)	

*Depth of primary tumor invasion*						
≤ 1/2	82.9%	Ref	0.001	77.8%	Ref	0.001
> 1/2	65.1%	1.4 (1.1.1.71)		58.2%	1.35 (1.06.1.67)	

*Histological grade*						
≤ G2	82.2%	Ref	0.001	78.5%	Ref	0.001
> G2	53.4%	1.55 (1.15.1.96)		47.2%	1.7 (1.19.2.31)	

*LNR*						
≤ 40%	82.6%	Ref	0.001	79.2%	Ref	0.001
> 40%	59.1%	1.4 (1.05.1.76)		56.6%	1.4 (1.05.1.76)	

*Lymphadenectomy*						
Pelvic	77.1%	Ref	0.71	71.1%	Ref	0.56
Pelvic + periaortic	74.2%	1.04 (0.71.1.35)		68.9%	1.03 (0.65.1.49)	

*Adjutant therapy*						
No	64.1%	Ref	0.031	58.9%	Ref	0.008
Yes	82.1%	0.78 (0.65.0.92)		79.1%	0.75 (0.55.0.95)	

*CALLY index*						
≥ 3	82.1%	Ref	0.012	76.4%	Ref	0.002
< 3	71.2%	0.86 (0.75.0.97)		64.2%	0.84 (0.74.0.95)	

*Note:* Kaplan–Meier analysis was used to estimate OS and RFS, and the difference between the outcomes for all variables in the two groups was compared using the log‐rank test.

**Figure 2:**
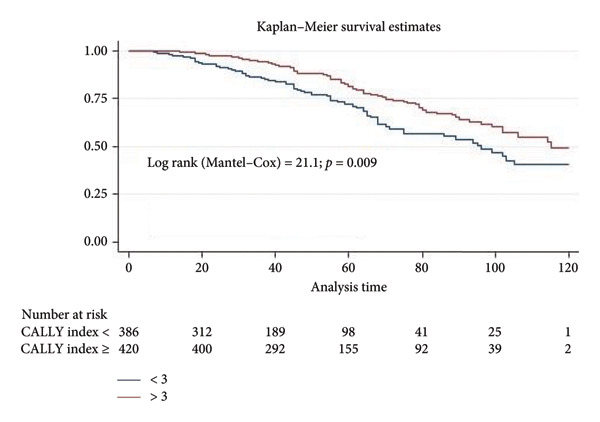
Kaplan–Meier overall survival analysis stratified by the CALLY index.

**Figure 3:**
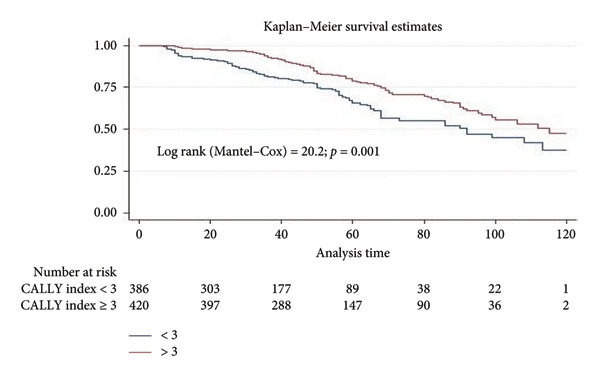
Kaplan–Meier RFS analysis stratified by the CALLY index.

The 5‐year OS and RSF rates were 76.9% and 70.5%, respectively. The 5‐year OS rate was significantly better in patients with a CALLY index ≥ 3 compared to < 3 ((82.1% vs. 71.2%) (log rank = 21.9, *p*: 0.009) (Figure 2). The 5‐year RFS rate in patients with a CALLY index ≥ three and < 3 was 76.4% and 64.2%, respectively, and this difference was statistically significant (log rank = 20.2, *p*: 0.001) (Figure 3).

### 3.2. Multivariate

VIFs were used to detect the presence of multiple collinearity between variables in the regression model (Supporting Table 1).

Cox multivariate analysis showed that, after adjusting for other variables, the CALLY index was significantly associated with the 5‐year OS and RFS rates. The 5‐year OS rate in patients with a CALLY index ≥ 3 was significantly better than in patients with a CALLY index < 3 (HR: 0.87, 95% CI: 0.78, 0.96, *p*: 0.001). The 5‐year RFS rate in patients with a CALLY index ≥ 3 was significantly better compared to those with a CALLY index < 3 (HR: 0.86, 95% CI: 0.78, 0.95, *p* = 0.001).

In addition, age, comorbidity, pathologic type, tumor size, FIGO stage, histological grade, depth of primary tumor invasion, adjuvant therapy, and LNR were significantly associated with OS and RSF in CC patients. While receiving was significantly associated with OS and RSF of CC patients, age ≥ 45 years, pathological type (nonsquamous), tumor size ≥ 3 cm, FIGO stage > Ib, histological grade > G2, and LNR > 40% were independent risk factors associated with poorer 5‐year OS and RFS rates in CC patients, while adjuvant therapy received was associated with improved 5‐year OS and RFS rates (Table 3).

**Table 3:** Predictors of OS and RFS in patients with CC based on the results of multivariate analysis.

Variable	OS			RSF		
	HR	95% CI	*p* value	HR	95% CI	*p* value
Age (year) (≥ 45 vs.< 45)	1.12	1.01.1.23	0.025	1.15	1.02.1.29	0.02
Pathologic type (nonsquamous vs. squamous)	1.53	1.09.1.96	0.004	1.58	1.1.1.99	0.004
Stage (FIGO) (< IB (1 or 2) vs. (> IB)	1.7	1.1.2.31	0.001	1.74	1.1.2.38	0.001
Histological grade (> G2 vs. *G* ≤ 2)	1.5	1.1.1.91	0.001	1.67	1.15.2.18	0.001
Adjutant therapy (positive vs. negative)	0.72	0.6.0.86	0.001	0.79	0.66.0.93	0.022
LNR (> 40% vs. ≤ 40%)	1.41	1.09.1.7	0.001	1.44	1.09.1.79	0.001
CALLY index (≥ 3 vs. < 3)	0.87	0.78, 0.96	0.006	0.88	0.77.0.99	0.001

## 4. Discussion

Although the proportion of radical hysterectomies has decreased in recent years after the laparoscopic approach to CC, this approach remains a common management approach for patients with CC, given its similar outcomes and complications to laparoscopy [16, 17].

In this multicenter retrospective cohort analysis, we found that the pretreatment CALLY index provides a significant independent predictive indicator for both OS and RFS in patients with early‐stage CC having radical hysterectomy. Patients with a CALLY index ≥ 3 had significantly higher 5‐year OS and RFS rates than those with a CALLY index < 3. Importantly, these relationships remained significant even after accounting for recognized clinicopathologic prognostic variables such as age, tumor stage, histological grade, tumor size, LNR, and adjuvant therapy.

Meta‐analyses have consistently shown that inflammatory and staging‐based indices provide prognostic value in CC. Elevated PLR is associated with reduced OS (HR = 1.77), PFS, and DFS, while high NLR before treatment predicts poorer OS (HR = 1.58) and increased recurrence and metastasis [8]. The FIGO 2018 staging system further underscores the prognostic impact of nodal metastasis, with ≥ 3 positive nodes and tumor size ≥ 4 cm significantly decreasing OS [18]. In comparison, findings from our multicenter cohort demonstrated that the CALLY index (cut‐off ≥ 3) independently predicted better 5‐year OS (HR = 0.87) and RFS (HR = 0.86), even after adjustment for conventional risk factors including FIGO stage, tumor size, and histological grade. Taken together, while PLR, NLR, and FIGO highlight systemic inflammation and anatomical disease burden, the CALLY index integrates host inflammatory status and nutritional reserve, offering complementary prognostic information that may better stratify early‐stage patients following radical hysterectomy.

The CALLY index has demonstrated superior prognostic value compared with the traditional TNM staging system, as evidenced by the CALLY–Epstein–Barr virus DNA index in nonmetastatic nasopharyngeal carcinoma, which integrated inflammatory, immune, and viral load parameters to enhance risk stratification beyond TNM alone [19] and by a CALLY index–based nomogram in non–small‐cell lung cancer, which combined CALLY index with clinical factors to more accurately predict 3‐ and 5‐year OS, outperforming the conventional TNM classification in prognostic resolution and clinical applicability [20], both providing more accurate survival predictions. In gynecology, a study including 190 patients in the training cohort and 120 in the validation cohort (a total of 310 patients) with epithelial ovarian cancer found that a CALLY index ≥ 3 was associated with significantly better OS and disease‐free survival (DFS), making it a novel prognostic biomarker after surgery [10]. Similarly, in breast cancer, a study of 174 patients showed that a CALLY index ≥ 2.285 was associated with better OS and DFS, with TNM stage and the CALLY index confirmed as independent prognostic factors, confirming that the CALLY index can serve as a reliable preoperative biomarker to guide postoperative risk stratification and identify patients who may benefit most from adjuvant therapy [15]. In a multicenter retrospective cohort of 657 esophageal cancer patients undergoing esophagectomy, a higher CALLY index (> 2.55) was independently associated with better OS (HR = 0.55) and DFS (HR = 0.51), consistent in both training and validation cohorts. Incorporating CALLY index into prognostic models significantly improved predictive accuracy (AUROC for OS: 0.719 · 0.752; DFS: 0.745 · 0.788), confirming its value as a cost‐effective tool superior to TNM staging alone for risk stratification and treatment planning [21]. In 318 patients with esophageal squamous cell carcinoma undergoing radical resection, a low CALLY index was associated with significantly worse 5‐year cancer‐specific survival (CSS) and remained an independent predictor (HR = 0.368). Across TNM stages I–III, higher CALLY index scores predicted better survival, and a CALLY index‐based recursive partitioning analysis model outperformed traditional TNM staging in prognostic accuracy (*p* < 0.001), demonstrating that integrating CALLY index with TNM can improve preoperative risk stratification [14].

Previous studies indicate that digestive system cancers remain a leading cause of cancer‐related mortality and have emerged as a promising prognostic marker. A meta‐analysis of 7951 patients showed that a lower CALLY index was significantly associated with worse outcomes across all survival endpoints, including OS (HR = 1.97), DFS (HR = 2.09), RFS (HR = 1.46), and CSS (HR = 2.46). It was a strong predictor of OS in surgical patients (HR = 2.01) [22]. Another systematic review of gastrointestinal malignancies reported that low CALLY index significantly predicted poorer OS (HR = 1.89, 95% CI: 1.72–2.08) and progression‐free survival (HR = 1.62, 95% CI: 1.44–1.81), with subgroup analyses showing worse OS in pancreatic cancer (HR = 1.77), cholangiocarcinoma (HR = 2.07), colorectal liver metastases (HR = 1.67), gastric cancer (HR = 1.88), colorectal cancer (HR = 2.28), hepatocellular carcinoma (HR = 1.65), and esophageal cancer (HR = 2.13) [23]. A meta‐analysis of 2829 gastric cancer patients demonstrated that high CALLY index was an independent favorable prognostic factor for OS and RFS, with better 5‐year survival and a higher proportion of early‐stage (T1) tumors. In contrast, low CALLY index was associated with lymph node metastasis, lymphovascular invasion, postoperative complications, and the need for adjuvant chemotherapy [11]. In other types of cancers, CALLY index also showed promising results. In a retrospective study of 199 HCC patients, the CALLY index, alpha‐fetoprotein (AFP), N/CRP, SII, ALBI, NLR, and AST/ALT ratio were all significantly associated with treatment response and survival, with AFP showing the highest predictive performance (AUC = 0.651) and the CALLY index being the second strongest predictor (AUC = 0.649), confirming its value in predicting HCC progression and outcomes [13]. In 201 ampullary carcinoma patients undergoing pancreaticoduodenectomy, a low CALLY index was independently associated with poorer cancer‐specific survival, confirming its value as a prognostic biomarker [24].

Three major biological mechanisms can explain the prognostic significance of the CALLY index. First, albumin, synthesized in the liver, serves as a central marker of nutritional status and systemic inflammation. Adequate serum albumin reflects sufficient protein reserves necessary for maintaining physiological resilience, supporting wound healing, immune competence, and tolerance to surgical or chemotherapeutic stress. Low serum albumin can result from malnutrition, inflammation‐induced hepatic dysfunction, and nutrient depletion caused by tumor protein consumption, particularly in gastric cancer, leading to hypoalbuminemia. Low albumin levels are associated with impaired drug distribution and metabolism, increased chemotherapy toxicity, higher rates of postoperative complications, and overall lower survival outcomes in cancer patients [11, 22, 23]. Second, CRP, an acute‐phase protein primarily induced by cytokines, such as interleukin (IL)‐6, tumor necrosis factor‐alpha (TNF‐α), and IL‐1β, reflects systemic inflammation and contributes to a tumor‐promoting microenvironment. Elevated CRP supports cancer progression by facilitating angiogenesis, invasion, metastasis, and immunosuppression. Chronic inflammation, characterized by elevated CRP levels, induces oxidative stress through the production of reactive oxygen and nitrogen species, resulting in DNA damage, chromosomal instability, and increased mutation rates. These factors collectively contribute to genomic instability and tumor aggressiveness. Moreover, CRP directly suppresses antitumor immunity by inhibiting CD8+ T‐cell activation via the FcγRIIb–p38MAPK–ROS signaling pathway, creating an immunosuppressive tumor microenvironment and worsening prognosis [11, 22, 23]. Third, lymphocytes, which include T‐cells, B‐cells, and natural killer cells, play a crucial role in both adaptive and innate immune responses against tumors. T lymphocytes directly destroy cancer cells, while B‐cells release cytokines such as IFN‐γ and TNF‐α to eliminate tumors. Natural killer cells, on the other hand, kill tumor cells without requiring antigen presentation. Low lymphocyte counts can result from nutrient deprivation in the tumor microenvironment, particularly deficiencies in glucose and amino acids. Low lymphocyte counts indicate impaired immune surveillance, which correlates with poor oncological outcomes across multiple malignancies [22, 23, 25].

Our study found that older age (≥ 45 years), nonsquamous histology, larger tumor size (≥ 3 cm), higher histological grade (> G2), LNR > 40%, and FIGO stage > Ib were all significantly associated with OS and RFS in early‐stage CC patients. These findings are consistent with previous meta‐analyses of the predictive value of these variables in the literature [26, 27]. In terms of adjuvant therapy, while our group showed increased OS and RFS, a meta‐analysis found no meaningful survival advantage in early‐stage CC [28].

The current study has various strengths. To the best of our knowledge, this is the first study to look at the predictive value of the CALLY index in patients with early‐stage CC. By focusing on a homogeneous population of patients who received standardized surgical therapy, we reduced potential confounding factors and increased the reliability of our findings. Furthermore, we assessed both OS and RFS to provide a full overview of long‐term outcomes. This study has limitations that should be recognized. First, the generalizability of our findings may be limited, as this study was conducted in a specific population with distinct characteristics, and its results may not apply to other populations. Second, due to the retrospective design of the study, we were unable to assess the effect of several key variables, such as molecular profiling and changes in the definition and assessment of various variables and markers over the 10‐year study period, which may have influenced the study results. Third, some potential confounding factors, including comorbidities and preoperative treatments, were not fully controlled for, which may have affected the observed associations.

## 5. Conclusion

In this study, the pretreatment CALLY index demonstrated independent prognostic value for both OS and RFS after adjustment for established clinicopathologic covariates in women with early‐stage CC treated with Type II/III radical hysterectomy. By integrating markers of systemic inflammation, nutritional status, and immune competence and being readily obtainable at low cost, the CALLY index may complement conventional risk factors to refine individualized postoperative risk stratification and follow‐up planning.

**Nomenclature**
AFP: Alpha‐fetoprotein
AUC: Area under the curve
BMI: Body mass index
CC: Cervical cancer
CALLY: C‐reactive protein–albumin–lymphocyte
CI: Confidence interval
CRP: C‐reactive protein
CSS: Cancer‐specific survival
DFS: Disease‐free survival
FIGO: International Federation of Gynecology and Obstetrics
HCC: Hepatocellular carcinoma
HR: Hazard ratio
IL: Interleukin
LNR: Lymph node ratio
NLR: Neutrophil‐to‐lymphocyte ratio
OS: Overall survival
PFS: Progression‐free survival
PLR: Platelet‐to‐lymphocyte ratio
RFS: Recurrence‐free survival
ROC: Receiver operating characteristic
STROBE: Strengthening the Reporting of Observational Studies in Epidemiology
TNF‐α: Tumor necrosis factor‐alpha
TNM: Tumor–node–metastasis

## Conflicts of Interest

The authors declare no conflicts of interest.

## Author Contributions

M.B., A.T., M.S.A., and A.A.: responsible for the study design, data analysis, findings interpretation, coding, writing the report, and preparing the manuscript. M.B., A.T., M.S., and S.G. acted as advisors and supervisors, providing crucial guidance and constructive feedback to improve the quality of the research. A.A., Y.T., M.H., A.T., A.A.G., and M.B. assisted by reviewing, editing, and approving the final version of the manuscript.

## Funding

The authors received no specific funding for this work.

## Supporting information


**Supporting Information** Table 1: Results of the collinearity analysis between variables.

## Data Availability

The data that support the findings of this study are available on request from the corresponding author. The data are not publicly available due to privacy or ethical restrictions.
