# Supplementary material for: Pretreatment CRP–Albumin–Lymphocyte (CALLY) Index as a Prognostic Biomarker of Survival and Recurrence‐Free Survival in Patients With Early‐Stage Cervical Cancer After Radical Hysterectomy: A Multicenter Retrospective Cohort Study
Source: Obstet Gynecol Int. 2026 Feb 2;2026:6137796. doi: 10.1155/ogi/6137796 (PMC12862499; doi:10.1155/ogi/6137796)
Supplement: Supplementary file 1 — Supporting Information Table 1: Results of the collinearity analysis between variables. [file OGI-2026-6137796-s001.docx]

| Model / variable | B |  | t | P value | Tolerance | VIF |
| --- | --- | --- | --- | --- | --- | --- |
| constant | 27.7 | 56.1 | 0.51 | 0.58 | - | - |
| Age | 0.52 | 0.27 | 1.97 | 0.055 | 0.56 | 1.81 |
| Comorbidity | 0.57 | 0.49 | 1.16 | 0.21 | 0.19 | 5.26 |
| Pathologic type | -0.33 | 0.37 | -0.89 | 0.54 | 0.64 | 1.55 |
| Stage (FIGO) | 0.17 | 0.52 | 0.32 | 0.77 | 0.22 | 4.4 |
| Depth of primary tumor invasion | 0.58 | 0.47 | 1.23 | 0.19 | 0.18 | 5.33 |
| Histological Grade | 0.17 | 0.2 | 0.84 | 0.40 | 0.38 | 2.64 |
| Adjutant therapy | 0.28 | 0.37 | 0.76 | 0.45 | 0.77 | 1.3 |
| LNR | 0.17 | 0.32 | 0.51 | 0.61 | 0.45 | 2.21 |

Table 1: Results of collinearity analysis between variables
